# Contribution of Corticospinal Tract and Functional Connectivity in Hand Motor Impairment after Stroke

**DOI:** 10.1371/journal.pone.0073164

**Published:** 2013-09-27

**Authors:** Charlotte Rosso, Romain Valabregue, Yohan Attal, Patricia Vargas, Marie Gaudron, Flore Baronnet, Eric Bertasi, Frédéric Humbert, Anne Peskine, Vincent Perlbarg, Habib Benali, Stéphane Lehéricy, Yves Samson

**Affiliations:** 1 Centre de Recherche de l’Institut du Cerveau et de la Moelle Epinière (CRICM), Université Pierre et Marie Curie, Paris, France; 2 Inserm, U975; CNRS, UMR 7225, Paris, France; 3 COGIMAGE, CRICM, Paris, France; 4 APHP, Urgences Cérébro-Vasculaires, Hôpital Pitié-Salpêtrière, Paris, France; 5 IFR49, DSV/I2BM/NeuroSpin, Université Paris 11, Gif-sur-yvette, France; 6 Institut du Cerveau et de la Moelle épinière, Centre de Neuro-Imagerie de Recherche (CENIR), Paris, France; 7 Service de Neurologie, Centre Hospitalo-Universitaire, Tours, France; 8 NeuroImagerie Cognitive, INSERM U562, CEA NEUROSPIN, Saclay, France; 9 AP-HP, Service de Médecine Physique et Réadaptation, Hôpital Pitié-Salpêtrière, Paris, France; 10 Laboratoire d’Imagerie Fonctionnelle, INSERM UMR_S678, Hôpital Pitié-Salpêtrière, Paris, France; 11 APHP, Service de Neuroradiologie, Hôpital Pitié-Salpêtrière, Paris, France; Biomedical Imaging Lab, Agency for Science, Singapore

## Abstract

**Background:**

Motor outcome after stroke is associated with reorganisation of cortical networks and corticospinal tract (CST) integrity. However, the relationships between motor severity, CST damage, and functional brain connectivity are not well understood. Here, the main objective was to study the effect of CST damage on the relationship between functional motor network connectivity and hand motor function in two groups of stroke patients: the severely (n=8) and the mildly impaired (n=14).

**Methods:**

Twenty-two carotid stroke patients with motor deficits were studied with magnetic resonance imaging (MRI) at 3 weeks, at 3 and 6 months. Healthy subjects (n=28) were scanned once. The CST injury was assessed by fractional anisotropy values. Functional connectivity was studied from a whole-hand grip task fMRI in a cortical and cerebellar motor network. Functional connectivity indexes were computed between these regions at each time point. The relationship between hand motor strength, ipsilesional CST damage and functional connectivity from the primary motor cortex (M1) was investigated using global and partial correlations.

**Findings:**

In mildly impaired patients, cortico-cortical connectivity was disturbed at three weeks but returned to a normal pattern after 3 months. Cortico-cerebellar connectivity was still decreased at 6 months. In severely impaired patients, the cortico-cortical connectivity tended to return to a normal pattern, but the cortico-cerebellar connectivity was totally abolished during the follow-up. In the entire group of patients, the hand motor strength was correlated to the ipsilesional functional connectivity from M1. Partial correlations revealed that these associations were not anymore significant when the impact of CST damage was removed, except for the ipsilesional M1-contralateral cerebellum connectivity.

**Conclusion:**

Functional brain connectivity changes can be observed, even in severely impaired patients with no recovery. Upper limb function is mainly explained by the CST damage and by the ipsilesional cortico-cerebellar connectivity.

## Introduction

Motor outcome after stroke is associated with the cerebral reorganisation of local and remote cortical areas, leading to restore a functionally efficient motor network [1–3]. Several longitudinal fMRI studies have described time-related changes of brain activation during the recovery of the paretic hand [4–17]. These studies and three recent meta-analyses [18–20] have shown that, in patients with good recovery, the activation pattern during paretic hand movement tended to return toward the original state. In contrast, in patients with poor recovery, the abnormal activations persisted. On the other hand, motor outcome or cerebral reorganisation seems also to depend on the location of the infarct lesion, especially the damage in the corticospinal tract (CST) [2,6,21–31]. The relationship between longitudinal functional changes and CST integrity has been assessed in only a few studies [4,6,13]. Many of these studies included only small subcortical lesions, and nearly all of them included patients who had already well recovered voluntary hand function [4,5,7,8]. Few studies have included severely impaired patients with no movement of their affected hand [6,12,13,17]. Conversely, the extent to which these results could be applied in patients with severe motor deficits is not well defined. However, severely impaired patients may provide useful data concerning the relationship between descending fibers integrity and longitudinal fMRI changes in cortical regions and in the cerebellum.

More recently, previous studies have used a connectivity-based approach to understand reorganisation processes in stroke [32–36]. Effective and functional connectivity could reflect the interaction between two regions that can be abnormal in patients with residual functional impairment even in spite of normal motor-related activations [1,3,37,38]. A recent study has investigated the impact of CST integrity on functional connectivity using resting state fMRI [32]. The authors showed that upper limb function was associated with CST integrity and inter-hemispheric connectivity in a motor network. They underlined the difficulty in disentangling the relative contribution of functional connectivity and CST damage on the motor performance of the affected hand.

Overall, these studies have highlighted the close relationship between the severity of hand motor impairment, the anatomical damage of the motor network, and the changes in the brain activity or functional connectivity, but few have integrated these informations together. Moreover, even though the role of some cortical areas is clear in the recovery process (essentially the ipsilesional primary motor cortex), their relative and independent contributions, removing the effect of the descending pathway injury, have not been extensively studied, including the contribution of the cerebellum. For the latter, two studies using resting-state [39] and task-based fMRI [40] have demonstrated that the functional interaction between the ipsilesional M1 and the contralesional cerebellum was decreased in stroke patients. However, these studies did not investigate the contribution of this dysfunctional cortico-cerebellar interaction in the hand motor deficit.

To this aim, we conducted a multimodal MRI study to analyze the following: (1) first, to characterise CST injury and the longitudinal changes in brain functional connectivity in two groups of patients with different outcomes: the severely (no movement of the paretic hand) and mildly impaired (able to move the paretic hand) stroke patients; and (2) second, to disentangle the relationship between the CST injury, the changes in the functional connectivity of the motor network, and the affected hand function.

Our hypotheses were as follows: (1) the severity of hand motor impairment is related to the extent of the CST damage [21–31]; (2) the hand motor impairment may be explained by some dysfunctional interactions in the cortico-cortical [33–35] and cortico-cerebellar motor networks [39,40] and (3) the association between hand motor impairment and the dysfunctional interactions in the motor network could be a reflection of its association with CST integrity [32].

## Materials and Methods

### Population

Fourty ischemic stroke patients and 28 healthy volunteers were prospectively recruited between January 2010 and January 2012. Patients were longitudinally examined at three time points: three weeks (V1), three months (V2) and six months (V3) after stroke onset. Inclusion criteria were as follows: (1) a first-ever ischemic stroke, (2) an initial MRI was performed within twelve hours of stroke onset and confirmed an ischemic lesion in the middle cerebral artery territory, and (3) a neurological deficit score of ≥1 on the National Institutes of Health Stroke Scale (NIHSS) in the motor items. Patients who met the following criteria were not included: (1) younger than 18 years old or under the care of a legal guardian, (2) functionally dependent before the stroke (modified Rankin score > 2), (3) displaying severe white matter lesions (Fazekas score > 2), or (4) addicted to alcohol or drugs, or diagnosed with a life-threatening pathology that would potentially limit the 6-month follow-up visit. We subsequently excluded 18 patients because they did not complete the entire protocol within three visits (n=12), failed the fMRI task because of head motion (n=4), or experienced difficulty in understanding the task (n=2). Therefore, only 22 patients were ultimately included in the study. These 22 patients were then divided in two groups based on their motor outcome at V1, i-e their ability to move the paretic hand: (1) a mildly motor impaired group (M group), including patients who could perform a fist closure movement with the paretic hand at V1 (n=14); and (2) a severely motor impaired group (S group), including the patients with complete motor hand deficit at V1 (n=8). All patients were given standard physiotherapy according to their deficits.

Healthy volunteers were recruited of the same age, handedness and gender compared to the patients. They underwent clinical assessment and multimodal MRI at a single time point. The study was approved by the local ethic committee of the Pitié-Salpêtrière Hospital in 2009. Written informed consent was obtained from each participant or from a legal proxy/family member if the patient had severe language disturbances or neglect.

### Clinical examination

The motor function of the patients was evaluated upon admission to the hospital (<12 hours, D0), at day seven (D7), and at each visit of the research protocol (V1, V2, V3) by using the motor upper limb capacity of the NIHSS score, which included items 5a or 6a depending on the side of the paretic hand (NIHSS-mu, up to 4 for a complete monoparesia) [41]. In addition, the maximal hand grip strength (mGS) was recorded three times and averaged at each visit (V1, V2, V3) using a specific device (MIE, Medical Research Ltd., (http://www.mie-uk.com/pgripmyo/index.html, please see supporting information file S1 for a more detailed description of the device). The mGS ratio was calculated as the mGS of the affected hand divided by the mGS of the unaffected hand. The Edinburgh Handedness Inventory (EHI) was performed to establish the laterality manual coefficient before the stroke event.

Healthy volunteers were tested using the maximal grip strength ratio (dominant vs. non dominant hand) and the EHI.

### MRI

All images were acquired with a 3T Siemens Trio MR Scanner. The MRI protocol included anatomical and functional sequences at each visit. A 12-channel head matrix coil was used. Head movements were restricted with foam pads. Subjects received instructions through sound-attenuating headphones. Patients were filmed with a camera during scanning in order to monitor mirror movements and to control the task.

#### Anatomical imaging

The MRI protocol included 3D-T1-SPGR (TR=2.3 s; TE=4.18 ms; flip angle=9°; TI=900 ms; matrix=240x256; voxel size=1x1x1 mm^3^; 176 slices), and spin-echo echo-planar diffusion tensor imaging (TR=10 s, TE=87 ms, FOV=256x256 mm^2^, slice thickness= 2 mm, 60 slices, 30 gradient encoded directions with a b-value of 1000 s/mm^2^ and 6 non diffusion-weighted volumes).

#### Functional MRI

BOLD contrast images were acquired using an echo-planar pulse sequence (TR=3 s, TE=25 ms, flip angle=90°, matrix=100x100, voxel size= 2x2x2.5 mm^3^, 47 volumes+ 6 volumes of dummy scans).

The motor task was a block-design, self-paced whole-hand grip task in which the subjects held an MRI-compatible device in their hand. This device was connected to a pressure transducer, which recorded and monitored the subject’s performances (frequency of the hand grip) during scanning (see supporting information file S1 for a more detailed description of this device). Self-paced condition was chosen instead of an imposed rate since both movement conditions activated a common motor network [42] and self-paced is less stress-full, requires less attention and probably leads to less head motions for the more impaired patients. Instructions were provided with auditory cue words as follows: “action” was used to initiate the alternation of hand squeezing and releasing the grip device until hearing the command “stop”. In severely motor impaired patients who were unable to move the paretic hand, the instructions were to attempt to move during the activation periods and to rest during the rest periods, as they did with the non-paretic hand. These instructions were similar to the instructions given in previous studies [12,13]. Only patients that understood the instructions and performed the task correctly with the unaffected hand were retained in the study. Careful attention was paid for every patient in order not to move the elbow and shoulder simultaneously with the hand. No mirror movement was noted. The paradigm consisted of 3 blocks of activation alternating with 4 blocks of rest. Each block lasted 20 seconds. Each hand was analysed in a separate run. All subjects were trained once for each hand to perform this motor task prior to entering the MR scanner device.

### Image processing

#### Anatomical images

Fractional anisotropy (FA) values in the CST were computed at V1 by using a CST template on each subject’s normalised FA maps as described previously [43]. DTI image processing was then carried out using FSL software tools from the FMRIB Software Library (University of Oxford, UK; FSL, v. 3.3. http://www.fmrib.ox.ac.uk/fsl). Diffusion images were corrected for eddy current distortions, and FA maps were generated using FDT (FMRIB’s Diffusion Toolbox) [44]. All pre-processed FA maps were normalised in Montreal Neurological Institute space (MNI http://www.bic.mni.mcgill.ca). For each normalized FA map, we performed a careful visual check for misregistration. To verify any potential residual errors, we computed the averaged FA maps in both healthy subjects and patients. Next, FA maps were compared by merging these maps with the CST template (see Figure S1 in Information S1). The CST template (obtained from histological data available at http://www.fz-juelich.de [45]) was superimposed onto each subject’s normalised FA map. The mean FA values were measured in the ipsilesional and contralateral CST. Segmentation of the CST template was employed because a comparison between this approach and tract-specific analyses using probabilistic tractography showed similar correlations with the behavioral measurements and a better correlation with the BOLD signal in a preliminary study [43].

Infarct volumes were delineated on the FLAIR images at V1 in the normalised space by interactive manual outlining using the MRICron software (http//:www.cabiatl.com/mricro/mricron). Periventricular white matter was visually assessed as damaged by a trained neurologist if the infarct lesion overlapped with the corona radiata or the centrum semi-ovale.

#### Functional images

Functional images were processed using SPM8 (http://www.fil.ion.ucl.ac.uk/spm/) and Matlab. All volumes were realigned to the first fMRI volume and co-registered with the anatomical images. An additional toolbox (ArtRepair, http://cibsr.stanford.edu/tools/human-brain-project/artrepair-software.html) was used to detect and correct for inter-scan fast motions. This type of motion correction has been previously used to better correct the data compared to the matching of mean head motions [46]. Volumes with a motion greater than 0.5 mm/TR, i.e., >1.5 mm (default parameters) were considered corrupted. If more than one-third of the functional sequences consisted of corrupted volumes with fast motion, the entire fMRI sequence was rejected for the analysis, and the patient was removed from this study. If less than one-third of the volumes were corrupted, then the functional sequence was repaired using linear interpolation (see supporting information file S1 for a more detailed description).

FMRI images were then normalised to the T1 template of SPM8 in the MNI space using the transformation matrix from anatomical images (3DT1) and smoothed using an isotropic 8 mm full-width at half-maximum Gaussian kernel. Each BOLD-time series’ normalisation was visually checked, and careful attention was paid to ensure that the regions of interest selected for the functional connectivity analysis were well positioned. In addition, we computed the average of the normalised BOLD-time series in patients and healthy subjects to compare the registration at specific z-coordinates of the ROIs (see Figure S2 in Information S1).

SPM contrast images for the first level for each subject were obtained using the general linear model, designed to detect brain activation related to the paretic hand movement with the default parameters (deconvolution with the canonical hemodynamic response function and high pass filtering fewer than 128 s). Functional images of left-sided stroke patients were flipped along the midsagittal plane so that all subjects could be treated as though the task had been performed with the left hand. Thus, the right hemisphere was the ipsilesional hemisphere (IL), related to the movement of the paretic hand. The left hemisphere was the contralesional (CL) hemisphere. The contrast images from each individual were then analysed using one-sample t-tests with age and mGS as confounding covariates in each group of patients. The height threshold was selected at p<0.01. The clusters were then considered significant at p<0.05, which was corrected for multiple comparisons.

**Figure 1:**
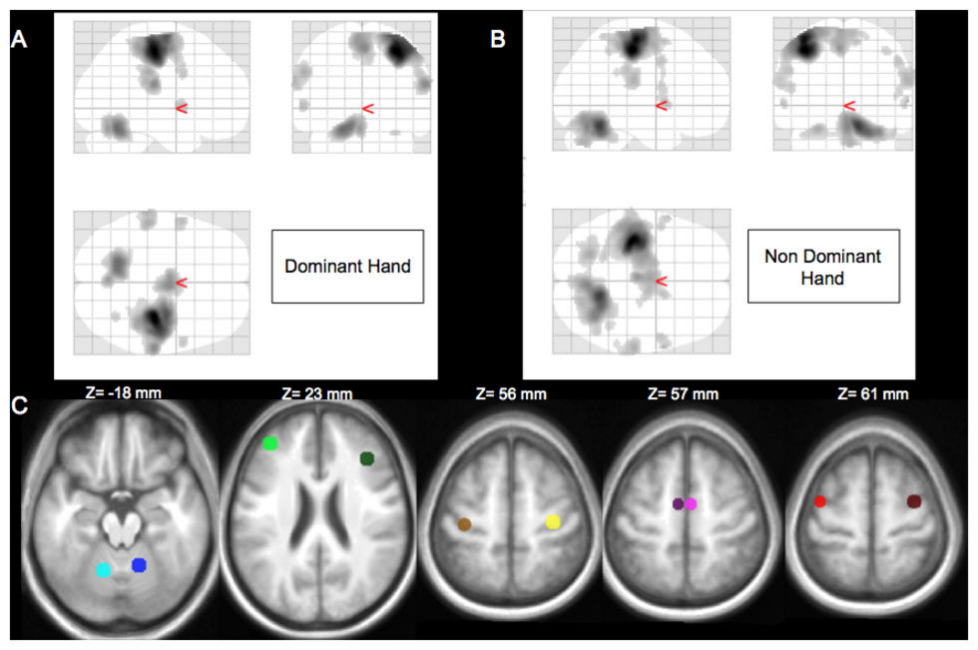
Determination of the motor network of interest in healthy subjects (n=14). SPM (T) contrast for the dominant (A) and the non-dominant (B) hand movement. The results are displayed on a ‘glass brain,’ shown from the right side (top left image), from behind (top right image), and from above (bottom left image). Clusters are significant at p < 0.05, corrected for multiple comparisons. (C) Overlap of the regions of interest (ROIs) on the MNI T1 template. Cerebellar ROIs are represented in blue (x = -14, y = -58, z= -18 mm for the left, and x = 14, y = 54, z=-18 for the right hemisphere); prefrontal cortex ROIs are represented in green (x = -38, y = 42, z= 22 mm for the left and x= 38, y = 30, z=24 for the right hemisphere); primary motor cortex ROIs are represented in yellow (x = -36, y = -22, z= 52 mm for the left, and x = 34, y=-20, z= 54 for the right hemisphere); supplementary motor area ROIs are represented in pink (x = -6, y = -6, z= 58 mm for the left, and x = 4, y = -6, z= 58 for the right hemisphere); and the lateral premotor cortex ROIs are represented in red (x = -40, y = -4, z= 58 mm for the left and x= 34, y=-4, z=60 for the right hemisphere). Right hemisphere is on the right side.

In order to define the motor network, healthy subjects were divided in two groups matched for age (p=0.94), gender (p=0.99) and handedness (p=0.64). The first group (n=14) was used to determine the motor network for subsequent region-of-interest (ROI) analyses (mean age: 54, IQR: 56-64; mean laterality coefficient: 0.75, IQR: 0.92-1 and sex ratio (M/F): 71%). The second group (n=14, referenced as HS group) was used as a control group for comparison with stroke patients. In the first group of healthy volunteers, Regions of Interest (ROIs) were defined using activation maps of the action vs. rest periods of the dominant and non-dominant hands separately, with age and mGS ratio as covariates (one-sample t-test, height threshold of p<0.001, cluster corrected at p<0.05 for multiple comparison, Figure 1A, 1B). There was no significant difference in the brain activations of the dominant vs. non-dominant hand in this sample of healthy subjects (see Figure S3 in Information S1). The ROIs were determined using the MarsBar toolbox (http://marsbar.sourceforge.net) as spheres with 6-mm radii centered on the peak coordinate of each of the following activated regions: primary motor cortex (M1), lateral premotor cortex (PMC, BA 6), prefrontal cortex (PFC, BA 9), associative parietal cortex (PAR, BA 40), putamen (PUT), supplementary motor area (SMA, BA 6) and contralateral cerebellum (CER) (Figure 1C). The right hemisphere was activated in response to movements of the non-dominant hand, which corresponded to the IL hemisphere in stroke patients. The left hemisphere was activated in response to the movement of the dominant hand, which corresponded to the CL hemisphere in stroke patients. However, two ROIs (PAR and PUT) completely overlapped with the lesions in six and twelve patients, respectively. Thus, these two ROIs were excluded from this network. The other ROIs did not present a significant overlap with the patients’ lesions (no more than 30%). The IL hemisphere network included the IL M1, IL PMC, IL PFC, IL SMA and the CL cerebellum. Conversely, the CL motor network included the CL M1, CL PMC, CL PFC, CL SMA and the IL cerebellum.

Functional connectivity between ROIs was analysed in each subject at each time point (V1, V2 and V3) with NetBrainWork software (GNU GENERAL PUBLIC LICENSE Version 3, 29 June 2007, https://sites.google.com/site/netbrainwork/). The fMRI time-series were initially corrected against physiological noise using CORSICA [47]. Accordingly, CORSICA was used to remove the fluctuations of no interest, which corrupted the BOLD signal, including rapid and slow head movements, physiological activity (breathing and heartbeat) and potential acquisition artifacts, to achieve structured noise reduction and to improve any subsequent detection and analysis of the signal fluctuations related to neural activity. Functional connectivity indexes were calculated as the correlations between any 2 ROIs in both hemispheres and between hemispheres (inter-hemispheric). Among all correlations, we focused the analysis on the inter-hemispheric homologous regions (i.e., M1 with M1, PMC with PMC, etc.) and the intra-hemispheric correlations between cortical areas and between cortical areas and the contralateral cerebellum.

### Statistical Analysis

#### Descriptive statistics

Descriptive statistics were performed using the median/mean and interquartile range (IQR) or standard deviations (SD). Comparisons of the proportions were performed using the chi-square test. For the between group analysis, quantitative variables were compared using non-parametric tests due to non-normal distributions and the small sample size in each group. The Kruskall-Wallis test was used to verify that the two groups of patients and healthy subjects were well matched for age, gender and handedness as well as between the two groups of patients for time delay between stroke and for each of the three visits.

In patients, the longitudinal analysis in each group of patients on the mGS and NIHSSmu, for which the distributions were not normal, was performed using the Friedman test for the factor session (V1, V2, V3). Post-hoc Wilcoxon tests were performed to compare between time points.

For the mean FA values in the CST, which followed a Gaussian distribution, a repeated-measures ANOVA was performed with the factor session as a within-subject factor and the factor group as a between-subject factor. Post-hoc t-tests were then used to compare the mean FA values at each time point (paired-t-tests) and between groups (independent t-tests). All of the descriptive statistical analyses were performed using MedCalc software (version 9.3.2.0, Mariakerke, Belgium), except for the Friedman test, which was performed using SPSS (version 20).

##### Characterisation of functional connectivity changes in stroke patients

For functional connectivity indexes, the correlation matrix displayed 45 correlations (10 within the IL motor network, 10 in the CL motor network and 25 inter-hemispheric correlations, including 5 inter-hemispheric homologous correlations). Between-region correlations were normalised using a Fisher transformation. Resulting Z-scores were thresholded to obtain significant correlations at p<0.05, FDR-corrected for multiple comparisons (False Discovery Rate) [48], which corresponded to a correlation coefficient of 0.245. Group differences of functional correlation indexes were inferred from the data using a fixed-effects group approach and a Bayesian group analysis with numerical sampling scheme [49]. Probability of differences between groups >0.9 was considered significant [50].

##### Correlation analysis between CST damage and functional connectivity with hand motor impairment in stroke patients

First, to study and examine the relationship between the imaging variables and hand motor function, we used global correlations between the functional connectivity indexes of the M1 and grip strength ratio using Spearman’s rank coefficient (with a 95% confidence interval) as well as between the FA values in the IL CST. Connectivity indexes in M1 were analysed in the IL motor network (IL M1-CER, M1-SMA and M1-PMC), the CL motor network (CL M1-PMC and M1-SMA), and between hemispheres (inter-hemispheric = IH, M1-M1). These correlations were also normalised using a Fisher transformation and were FDR-corrected for multiple comparisons. We further examined the partial correlation coefficients between grip strength ratio and functional connectivity indexes to remove the effect of changes due to FA in the CST. Partial correlation coefficients measure the degree of association between two variables (i.e., grip strength ratio and functional connectivity indexes from M1) while removing the effect of controlling random variables (i.e., FA values in the ipsilesional CST) [52]. The R^2^ value calculated using the global and the partial coefficients were compared to evaluate the relative weight of CST damage on the correlation between grip strength ratio and functional connectivity indexes. Before, since it has been previously reported that stroke patients could exhibit bilateral deficits in dexterity [51], we analyzed the z-scores of the mGS of the unaffected hand normalized against the mGS of the non-dominant hand in healthy subjects to demonstrate that the mGS of the unaffected hand was a valid form of measure. To achieve this, we used the following formula (mGS of the unaffected hand for each patient *minus* mean mGS of the non-dominant hand *divided by* the standard deviation of the mGS of the non-dominant hand in the healthy subjects). Next, these z-scores were analyzed against the null hypothesis using a one-sample-t-test. We found no significant difference at V1 (mean: -0.07, SD: 0.16; p: 0.06), V2 (mean: -0.22; SD: 0.15; p: 0.16) and V3 (mean: -0.34; SD: 0.21; p: 0.12).

Finally, to verify that these relationships were specific to the motor network, we performed exactly the same analysis using a visual network at V1 (including two ROIs in the right and the left primary visual cortices) as a control network.

## Results

### Population

The baseline and follow-up characteristics of the 2 groups of patients and healthy volunteers are presented in table 1 (and see table S1 in supporting information file S2 for individual characteristics). Age (p=0.16), gender ratio (M/F, p=0.2), and laterality quotient by the EHI (p=0.3) did not differ between the 3 groups. The median time delay between stroke and each of the three visits was identical in the two groups of patients (median, IQR at V1: 25 (15–35) *vs.* 28 days (22–34), p=0.9; median, IQR at V2: 93 (89–95) *vs.* 88 days (83–94), p=0.9; median, IQR at V3: 233 (204–254) *vs.* 213 days (196–221), p=0.95, for the mildly and severely impaired groups, respectively).

**Table 1:** Clinical characteristics of patients and healthy subjects.

Mean, IQR	S group n=8	M group n=14	HS group n=14
Age (years)	44 (35-53)	57 (45-64)	53 (42-64)
Sex ratio (M/F, %)	5/8 (62%)	8/14 (57%)	5/14 (36%)
EHI quotient	0.89 (0.87-1)	0.62 (0.670-1)	0.82 (0.91-1)
FA in damaged CST	0.268 (0.249-0.288)	0.381 (0.355-0.411)	0.378 (0.364-0.397)
FA in normal CST	0.370 (0.362-0.390)	0.398 (0.377-0.423)	0.413 (0.382-0.444)
mGS ratio			
V1	0 (0-0)	0.91 (0.72-1.05)	1.18 (1.09-1.31)
V2	0 (0-0)	0.92 (0.84-1.04)	
V3	0 (0-0)	0.93 (0.77-1.07)	

Values are the means and interquartile ranges (IQR). Abbreviations: M: mildly impaired patients, S: severely impaired patients, HS: healthy subjects, mGS: the maximal grip strength ratio of the affected hand, EHI: Edinburgh Handedness Inventory, FA: fractional anisotropy values, CST: corticospinal tract.

### Motor profile of patients

Mildly impaired patients exhibited mild motor deficits upon admission (mean NIHSSmu: 1.7, IQR: 1-3). There was an overall improvement in the NIHSSmu with time (p<0.0001). Post-hoc Wilcoxon tests showed that mildly impaired patients presented a rapid improvement in the NIHSSmu between the day of admission and day 7 (p<0.0005). The NIHSSmu returned to normal values (0) before the research protocol (V1), indicating that mildly impaired patients no longer exhibited proximal upper limb impairment. The mGS ratio did not improve with time (p: 0.91) but was smaller compared to healthy subjects at V1 (p<0.004), V2 (p<0.007) and V3 (p<0.003) indicating that there was a persistent and stable decrease in the strength of the affected hand during the 6-months follow-up period. Despite this lack of strength, the frequency of the paretic hand movements into the scanner was similar to those of the healthy subjects (median and IQR: 0.93 Hz, 0.58-1.29 *vs.* 0.92 Hz, 0.78-1.13).

Severely impaired patients demonstrated a different motor profile. Hand function did not recover at all because the NIHSSmu and the mGS ratio did not improve over time (see table 1). Patients were unable to move their paretic hand throughout the entire study.

### Anatomical damage

In the entire sample of patients, there was an over-all significant effect of the factor time on the mean FA values of the IL CST (F(1,20) : 9.53, p : 0.006). Post-hoc paired-T-tests revealed that the FA values in the entire sample of patients were similar between V1 and V2 (mean ± SD: 0.334 ± 0.059 vs. 0.331 ± 0.067, p : 0.63) but decreased between V2 and V3 (mean FA ± SD at V3 : 0.319 ± 0.071, p : 0.01).

**Figure 2:**
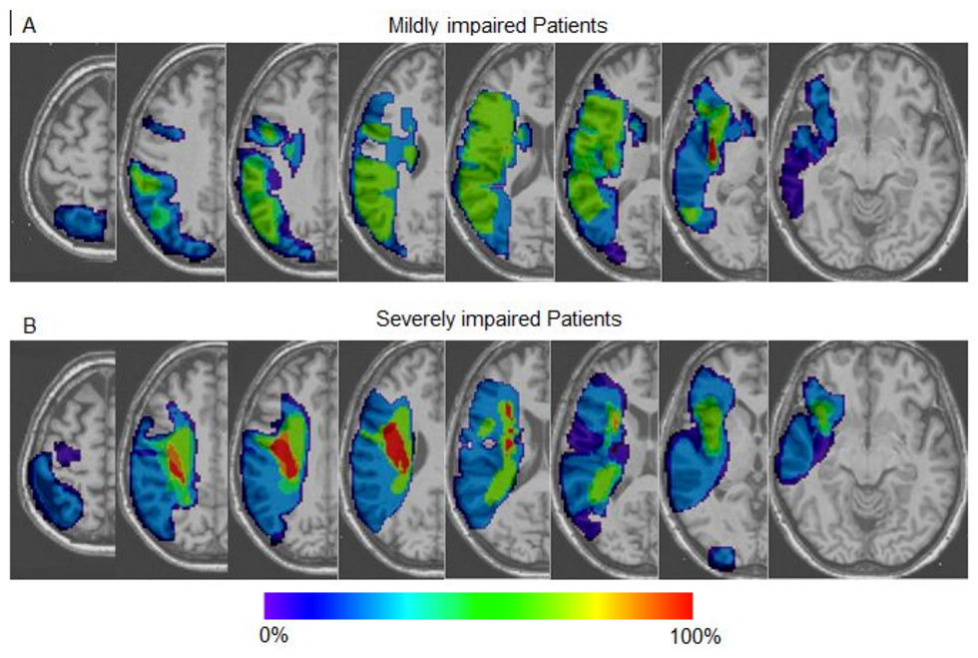
Localisation of infarction. Overlap of infarct lesions on a T1 anatomical template in (A) mildly impaired patients (n=14) and (B) severely impaired patients (n=8). Colour bar indicates the proportion of patients with infarction for each voxel.

Mildly impaired patients displayed smaller lesions (mean infarct volume 34 cm^3^; IQR: 2-51, p=0.007) than the severely impaired patients, less frequently involving the periventricular white matter (28.5%, 4/14, p=0.02) (Figure 2A). The CST was involved in 71% of patients (10/14) with a mild degree of damage. The FA values in the ipsilesional CST were significantly higher than those in the severely impaired group (p<0.0001, Table 1).

Extensive lesions (mean infarct volume 101 cm^3^; IQR: 59-155) involving the periventricular white matter were observed in 88% (7/8) of severely impaired patients (Figure 2B). The CST was affected in all patients. The FA values in the ipsilesional CST were markedly decreased compared to healthy subjects or mildly impaired patients (p<0.0001).

### Characterisation of functional connectivity changes over time in the motor network

**Figure 3:**
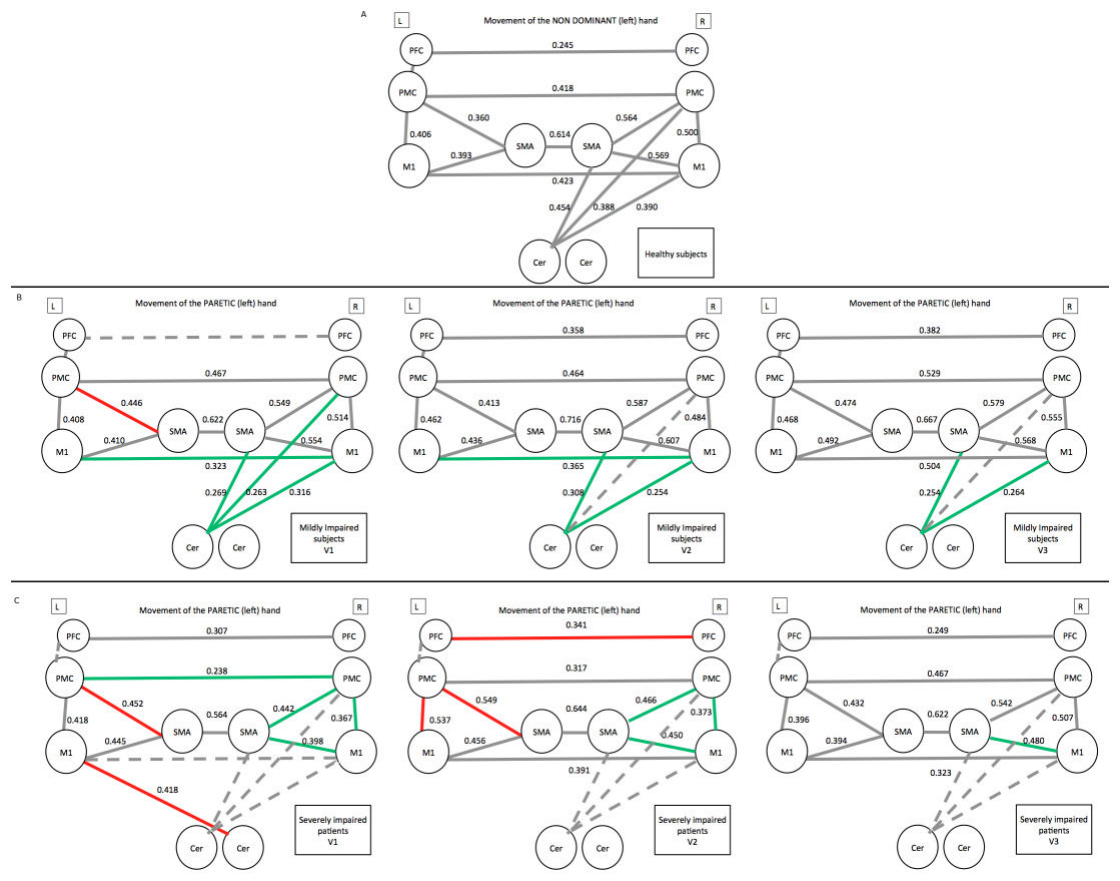
Functional connectivity during hand movements. (A) Movements of the left hand in healthy subjects, and of the paretic hand in (B) mildly impaired and (C) severely impaired patients. In patients, grey lines indicate that correlations were normal and identical to the healthy subjects, red lines indicate that correlations were increased, and green lines indicate that correlations were reduced compared with healthy subjects. Dotted lines indicate that the correlation is not significant in the group of patients. The level of the significant correlations is indicated near the corresponding line. Abbreviations: L: left, R: right, V1, V2 and V3: visit 1, 2 and 3.

Figures S1 and S2 (in Information S2) provide the functional activation maps at the group level for the motor task compared with rest for each group of patients and at each time point. Figure 3 presents the connectivity values for hand movements in healthy subjects and of the paretic hand in patients. In healthy subjects, correlations were significant in both hemispheres for M1-SMA, M1-PMC, PMC-SMA and between the right cortical regions (SMA, PMC and M1) and the contralateral cerebellum. Correlations were also significant between all inter-hemispheric homologous regions. In the ipsilateral hemisphere corresponding to the CL hemisphere in patients, the PFC-PMC correlation was also significant.

In the mildly impaired group at V1, the main differences included *reduced* correlations between cortical regions in the IL hemisphere and the contralateral cerebellum, reduced correlations between both M1s, and *increased* correlation between CL PMC-SMA in the healthy hemisphere. At V2, the CL motor network correlations returned to normal. At V3, IH correlation between M1s returned to baseline values, but reduced correlations between cortical regions in the IL hemisphere and the contralateral cerebellum persisted.

In the severely impaired group at V1, the main differences included reduced correlations between cortical regions in the IL hemisphere (M1-SMA, M1-PMC, PMC-SMA), reduced inter-hemispheric correlation between both PMCs and M1s, and the disappearance of the correlations between IL cortical regions and the contralateral cerebellum. Two correlations were increased in the CL hemisphere (CL PMC-SMA and CL M1 to IL cerebellum). Overall, decreased correlations in the IL hemisphere persisted at V2 as well as the increased correlation between CL PMC and SMA. In the CL hemisphere, there were also increased correlations between inter-hemispheric PFCs and CL PMC-M1. At V3, these correlation values returned to normal values, except for that of the IL M1-SMA correlation, which remained decreased. Correlations between IL cortical regions and the contralateral cerebellum remained not significant at all time points.

An additional cross-sectional analysis, which included 5 additional patients at V1, is presented in the supporting information file S2.

### Correlation analysis between CST damage and functional connectivity with hand motor function in stroke patients

CST damage in the entire group of patients was highly correlated with the grip strength ratio of the affected hand at V1 (rho: 0.853; 95%CI: 0.674-0.937, p: 0.0001) but also at the end of follow up at V3 (rho: 0.815; 95%CI: 0.553-0.909, p: 0.0001).

The global correlations were significant at V1 for the functional connectivity indexes in M1 in the IL hemisphere (IL M1- CL CER, IL M1-SMA and IL M1-PMC, Table 2). Only the IL M1- CL CER correlation remained significant at follow-up (p: 0.006 at V3). Grip strength ratio did not correlate with functional connectivity values in the CL motor network (p<0.6). Once the possible contribution of the CST damage on the functional connectivity was removed by partial correlation, only the IL M1-CL CER correlation remained significant. Nevertheless, the proportion of the variance of the grip strength ratio explained by the IL M1-CL CER functional connectivity (the R^2^ values) decreased from 52% to 15% after removing the effect of the CST damage on the global correlation. This partial correlation was still significant with the grip strength ratio at V3 (p=0.005). In addition, when the infarct volumes also accounted for the control covariate using the mean FA values, the R^2^ did not significantly decrease (R^2^: 15 *vs.* 12.6%, p: 0.83).

**Table 2:** Global and partial (removing the impact of the CST injury) correlations (with 95% CI) between grip strength ratio and functional connectivity in M1.

	Global correlation with the grip strength ratio	Partial correlation with the grip strength ratio
IL M1-CER functional connectivity	0.719* (0.427, 0.875)	0.388* (-0.040, 0.696)
IL M1-PMC functional connectivity	0.578* (0.207, 0.804	0.284 (-0.156, 0.630)
IL M1-SMA functional connectivity	0.476* (0.068, 0.748)	0.372 (-0.059, 0.696)
IH M1-M1 functional connectivity	0.350 (-0.084, 0.672)	0.119 (-0.319, 0.515)

Abbreviations: IH: interhemispheric, CL: contralesional hemisphere, IL: ipsilesional hemisphere, M1: primary motor cortex, PMC: premotor cortex, CER: cerebellum, SMA: supplementary motor area. *p<0.05.

In the control visual network, there was a decreased interaction between both occipital regions in the mildly and severely impaired patients compared to the healthy subjects (mean ± SD correlation in the mildly impaired patients: 0.618 ± 0.029; in the severely impaired patients: 0.630 ± 0.035 and in the healthy subjects: 0.705 ± 0.021). No significant difference was observed between the mildly and severely impaired patients. This functional connectivity index was correlated neither with the mGS ratio (p: 0.80), nor with the IL CST damage (p: 0.65).

## Discussion

The comparison between severely and mildly impaired patients showed several important differences. Patients with severe impairment presented 1) reduced cortico-cortical interactions in the damaged hemisphere and increased interactions in the contralesional hemisphere that tended to normalize with time despite a lack of recovery of hand motor function, 2) abolished connectivity between damaged cortical areas and the contralesional cerebellum that did not recover with time, and 3) severe CST damage. Conversely, mildly impaired patients had activation and functional interactions levels in the cortex similar to that of healthy subjects at the end of the follow-up period, but reduced cortico-cerebellar functional connectivity with moderate involvement of the CST. Upper limb function was highly correlated with CST integrity and functional connectivity of the damaged motor cortex. The main determinant of upper limb motor function was the amount of CST damage, followed by the ipsilesional M1 – cerebellum functional connectivity, which remained involved in hand motor impairment independently of the degree of CST damage.

### Characterisation of functional brain connectivity changes in severely and mildly impaired stroke patients

Mildly impaired patients exhibited transitory dysfunctional interactions between cortical areas but showed persistent reduction in functional connectivity between cortical motor areas and the contralateral cerebellum. In contrast, cortico-cerebellar functional connectivity was completely abolished in severely impaired patients. In these patients, reduction in cortico-cortical functional connectivity that was observed at 3 weeks returned to normal values at 6 months, except for a persistent decrease in M1-SMA interactions in the damaged hemisphere, in line with previous studies [33]. Overall, these results suggest that recovery of normal levels of functional interactions in the cortex is not necessarily associated with good recovery of motor function. Nevertheless, some of the transitory abnormalities of the cortico-cortical network observed in severely impaired patients, such as the increased interactions between both the PFCs at V2, might be a reflection of the attentional and motor planning demands because the subjects had attempted to perform the movement without success. In contrast, the absence of connectivity between the damaged motor areas and the contralateral cerebellum is associated with poor outcome and is probably a consequence of the disruption of descending tracts, such as the cortico-pontine tract, which travel through the corona radiata and the internal capsule [53–55].

### Correlations between anatomical damage, functional changes and hand motor function

In agreement with previous studies, we found that greater CST damage was globally associated with greater severity in hand motor deficits [22,23,26,28]. Here, we further investigated the link between CST damage, functional connectivity changes and motor impairment. The main results were the followings: (1) cortico-cortical functional connectivity during motor task in the damaged M1 correlated with grip strength of the affected hand, but this correlation was no longer significant when the contribution of the CST damage was removed; (2) functional connectivity of the undamaged motor cortex and between hemispheres did not correlate with affected hand function; and (3) the ipsilesional cortico-cerebellar functional connectivity correlated positively with the grip strength ratio, even when removing the contribution of the CST,

In contrast to the present findings, previous study reported that CST damage correlated better with inter-hemispheric than with functional connectivity in the damaged hemisphere [32]. This apparent disagreement may be due to differences in patients’ status during MRI acquisition: resting state in [32] versus movement performance here, as abnormal coupling between motor regions in stroke patients differed during movement and rest [34].

The partial correlation coefficient approach suggested that M1 – cerebellar functional connectivity played a role in hand grip strength recovery independently from the degree of CST damage. The importance of the cerebellum in motor function after stroke was first suggested by the observation of the cerebellar diaschisis in PET studies [56,57] and by the presence of a correlation between cerebellum activity and recovery in fMRI studies [14]. Connectivity changes between the damaged M1 and the controlesional cerebellum has already been reported in motor stroke patients [39,40] but the contribution of this M1-cerebellum interaction in the hand motor impairment was not described. Indeed, this may have potential therapeutic implications. For instance, the cerebellum may represent a potential target for Transcranial Magnetic Stimulation (TMS) that is currently being tested as a therapeutic tool in stroke patients in ipsi- or contralesional M1s [58,59]. In patients with mild or moderate CST damage, non-invasive brain stimulation techniques may focus on facilitating the cortico-cerebellar connectivity. Moreover, stimulation of the damaged primary motor region probably involves both transcallosal and cortico-cerebellar effects via the descending motor pathway. Stimulation of these descending tracts may provide an explanation to the observation that stimulation of the ipsilesional M1 improved better motor function than inhibition of the contralesional M1 [58]. Recently, Ziemann et al (2013) have also reported in healthy subjects an increased excitability of M1 when they targeted in a paired-associative stimulation protocol the cerebellum with the primary motor cortex [60].

### Limitations

There are several limitations to our study. First, although the total sample size was in the higher range of published longitudinal fMRI studies, the number of subjects in each subgroup was relatively low, limiting the intra-subgroup analysis. Secondly, we included severe stroke patients with no residual hand function. In these patients, it is difficult to ensure that subjects actually performed the task, as there was no motor output. BOLD signal changes depend on task performance but task performances reflect also the size and the location of the lesion [13]. We ensured that severely impaired subjects completed the task with the unaffected hand, demonstrating that they understood the instructions and suggesting that they actually tried to move the affected hand accordingly. In addition, the BOLD signal changes and functional connectivity evolved with time and returned to normal values in the cortex for the latter, despite the lack of movement of the affected hand. We speculated that the functional changes in this group were related to inefficient brain processes that were associated with plasticity (with no changes in behavior) or that application of the instructions for the motor task might require less effort with time. Thirdly, subjects were followed from 3 weeks to 6 months after the stroke, and changes may have occurred before and after this period of time that were not captured here. However, it seems unlikely that severely impaired patients would have regained hand function after this time [61]. Nevertheless, it would be interesting to determine if persistent dysfunction at 6 months would remain stable at 1 year. Another limitation is that motor reorganization after stroke is known to recruit other areas. These areas may be missed when using this approach where the configuration of the healthy subjects’ motor network to the patients is applied. Finally, it was not possible to include the putamen and the associative parietal cortex in the motor network analysis as these structures were severely damaged by the infarct in more than half of the patients. This would have directly impacted the BOLD signal changes. Therefore, in the analysis, we only included regions that were not entirely damaged by the infarct.

In conclusion, changes in functional brain connectivity were observed longitudinally in remote infarcted areas, even in severely impaired patients with no recovery. The main difference in the functional pattern between severely and mildly impaired patients concerned cortico-cerebellar functional connectivity between the damaged cortex and the contralesional cerebellum. Upper limb function in stroke patients was mainly explained by the CST damage and, to a lesser degree, by the cortico-cerebellar functional connectivity.

## Supporting Information

Information S1 **Supplementary material and methods.**
(DOC)

Information S2 **Supplementary results.**
(DOC)
