# Correction: Contribution of Corticospinal Tract and Functional Connectivity in Hand Motor Impairment after Stroke

**DOI:** 10.1371/annotation/1f472957-84b4-4251-9abb-3430469b14dd

**Published:** 2014-01-03

**Authors:** Charlotte Rosso, Romain Valabregue, Yohan Attal, Patricia Vargas, Marie Gaudron, Flore Baronnet, Eric Bertasi, Frédéric Humbert, Anne Peskine, Vincent Perlbarg, Habib Benali, Stéphane Lehéricy, Yves Samson

A reference was omitted from the reference list during the production process. There reference is: 53. Middleton FA, Strick PL (1994) Anatomical evidence for cerebellar and basal ganglia involvement in higher cognitive function. Science 266: 458-461.

In place of reference 53, some erroneous formatting text was published in the PDF. 

