# Supplementary material for: Contribution of Corticospinal Tract and Functional Connectivity in Hand Motor Impairment after Stroke
Source: PLoS One. 2013 Sep 27;8(9):e73164. doi: 10.1371/journal.pone.0073164 (PMC3785485; doi:10.1371/journal.pone.0073164)
Supplement: Information S1 — Supplementary material and methods. (DOC) [file pone.0073164.s001.doc]

**Supporting Information File S1 : Supplementary material and methods**

Hand grip device

The pinch grip device was generated by MIE Research Ltd. (<http://www.mie-uk.com/pgripmyo/index.html>). The pinch grip analyzer measures the hand grip pinch performance. The handle consists of a unique and lightweight but stiff aerospace alloy and is adjustable to accommodate all hand sizes and fixed flexion deformities. MIE's patented design overcomes any leverage effects when gripping the handle; thus, the measured force is the same irrespective of hand position. Therefore, the therapist is able to support the entire weight of the handle to administer tests on particularly frail subjects. Moreover, the system is able to directly connect to the serial multi-analyzer and is able to obtain an objective measurement of grip strength in Newtons.

MRI-compatible device for task monitoring

The fMRI motor task was a fist closure task, which was monitored using a pressure transducer that was positioned in the subject’s hand (1). This power grip consisted of two plastic molded handles that surrounded a rubber tube containing air, which was sufficiently long to lead to the control room via a waveguide. On the power grip side, the tube was sealed. On the control room side, the tube was plugged into a transducer that converted the air pressure into a voltage. Thus, when the subject squeezed the power grip, the air pressure inside of the tube increased as well as the tension output of the transducer. This system enabled the ability to monitor the frequency of the device when squeezed (provided by the Wellcome Trust Centre for Neuroimaging, London, UK). The analogue voltage was then digitalized via an analogue-digital converter (CED 1401, Cambridge Electronics, Cambridge, UK) and sent to Matlab (Mathworks, Natick, MA, USA), which ran a stimuli presentation script program using Cogent 2000 (Wellcome Trust Centre for Neuroimaging, London, UK).

(1) Schmidt L, Lebreton M, Cléry-Mélin ML, Daunizeau J, Pessiglione M. Neural mechanisms underlying motivation of mental versus physical effort. PLoS Biol. 2012; 10(2): e1001266. doi:  [10.1371/journal.pbio.1001266](http://dx.doi.org/10.1371%2Fjournal.pbio.1001266)

Quality control of the normalisation step for the fractional anisotropy (FA) maps and BOLD-time series in the MNI space

For each normalized FA map, we performed a careful visual check for misregistration. To verify any potential residual errors, we computed the averaged FA maps in both healthy subjects and patients (see figure S1). Next, these maps were compared by merging the maps with the CST template.

| 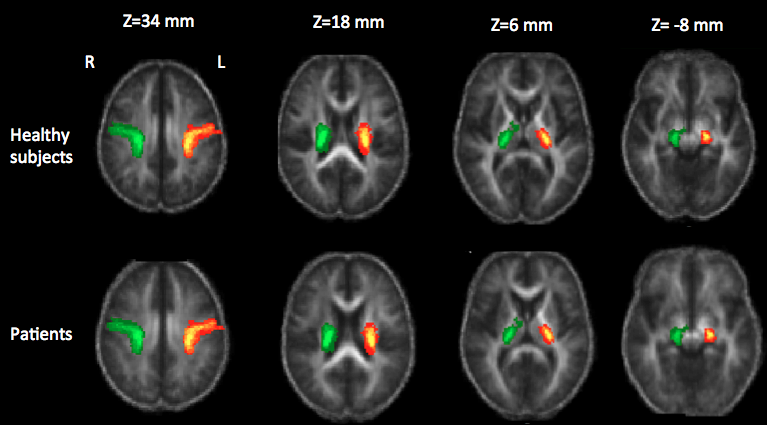 |
| --- |

Figure S1: Averaged FA maps in healthy subjects and patients. The CST template was merged onto the average FA map of each group (the orange and green colors represent the left CST and right/ipsilesional CSTs, respectively). R = right and L = left.

BOLD-time series’ normalisations were visually checked, and careful attention was paid to ensure that the regions of interest selected for the functional connectivity analysis were well positioned. In addition, we computed the average of the normalized BOLD-time series in patients and healthy subjects to compare the registration at specific z-coordinates of the ROIs (Figure S2).

| 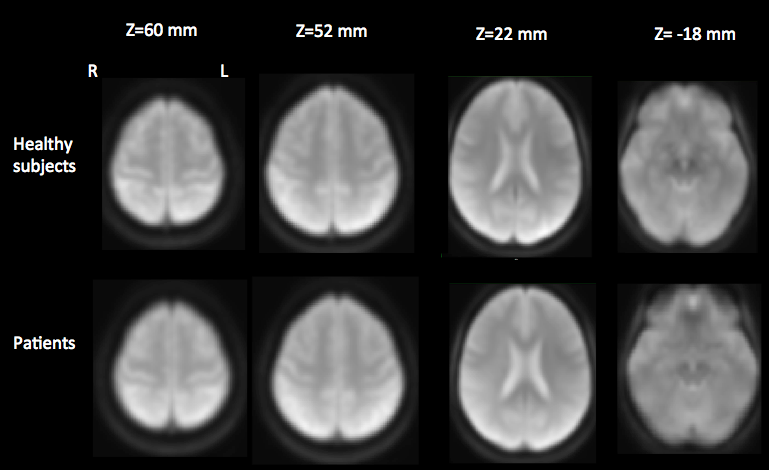 |
| --- |

Figure S2: Averaged BOLD-time series in healthy subjects and patients. The z-coordinates correspond to the location of the regions of interest for the functional connectivity analysis: z = 60 mm for the supplementary motor area and premotor cortex, z = 52 mm for the primary motor cortex, z = 20 mm for the prefrontal cortex and z = -18 mm for the cerebellum. No difference was visually detected in the location of the ROIs.

Motion correction : ArtRepair steps

The volume artifact program in ArtRepair (ArtRepair, http://cibsr.stanford.edu/tools/human-brain-project/artrepair-software.html) analyzes both global intensity and scan-to-scan movement after realignment and re-slicing of the EPI volumes. The graph shows the scan-to-scan movement. The vertical scale is represented as “mm/TR” (the rotation assumes that a voxel is 80 mm from the origin). The default threshold line was plotted at 0.5 mm/TR variation. A still subject would result in less variation compared to the scan-to-scan motion. The default thresholds of the programs used in the study estimated the outlier volumes and additional volumes to be de-weighted during estimation. The outlier volumes to be repaired were specified by red vertical lines. The outliers may also be caused by exceeding the intensity variation threshold or by exceeding the scan-to-scan motion threshold. The repair step would repair any volumes that were outliers at the current threshold. Moreover, we used the INTERP button as the best method of repair. This function fill values use a linear interpolation from the nearest unrepaired scans. The mean fill values reflect the mean scan of the run. The de-spike fill values use a linear interpolation of the immediately preceding and following scans regardless of whether they were repaired.

Brain activation maps for healthy subjects for both the dominant and the non-dominant hand

| 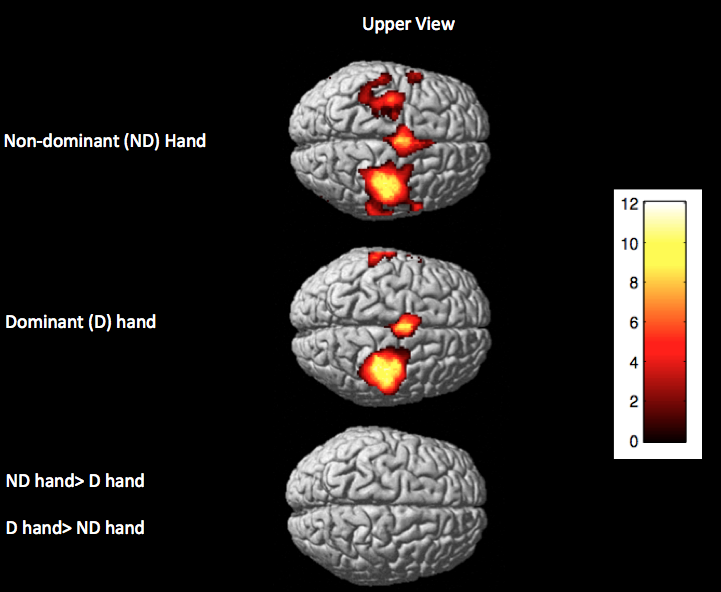 |
| --- |

**Figure S3:** Surface rendering of the fMRI activation maps for the contrast of the “non-dominant hand movement vs. rest” (upper row), “dominant hand movement vs. rest” (middle row) and the differences between “non-dominant and dominant hand movement” (lower row). The height threshold was 0.001, which was corrected at the cluster level for multiple comparisons (p: 0.05). The color bar indicates the t-statistics. There was no significant activated cluster in the contrast of the brain activations between the dominant and non-dominant hand movements.
