# Supplementary material for: Contribution of Corticospinal Tract and Functional Connectivity in Hand Motor Impairment after Stroke
Source: PLoS One. 2013 Sep 27;8(9):e73164. doi: 10.1371/journal.pone.0073164 (PMC3785485; doi:10.1371/journal.pone.0073164)
Supplement: Information S2 — Supplementary results. (DOC) [file pone.0073164.s002.doc]

**Supporting Information File S2 : Supplementary Results**

Individual characteristics of the patients

Table S1: Individual characteristics of the patients

Group 1 : severely impaired patients, group 2 : mildly impaired patients, M : male, F : female, R : right, L : left, mGS : maximal grip strength ratio, NIHSSmu : motor item for the paretic upper limb, V1 : first visit, V2 : second visit, V3 : third visit.

|  | group | age | sex | side | EHI | mGS  V1 | mGS  V2 | mGS  V3 | NIHSSmu  V1 | NIHSSmu  V2 | NIHSSmu  V3 |
| --- | --- | --- | --- | --- | --- | --- | --- | --- | --- | --- | --- |
| 1 | 1 | 57 | M | R | 0,91 | 0 | 0 | 0 | 4 | 4 | 4 |
| 2 | 1 | 35 | F | R | 0,83 | 0 | 0 | 0 | 4 | 3 | 2 |
| 3 | 2 | 64 | M | L | 0,67 | 1,24 | 1,49 | 0,92 | 0 | 0 | 0 |
| 4 | 2 | 54 | M | L | 0,83 | 1,08 | 1,35 | 1,06 | 0 | 0 | 0 |
| 5 | 2 | 40 | F | R | 1 | 0,69 | 0,77 | 1,05 | 0 | 0 | 0 |
| 6 | 1 | 48 | F | R | 0,92 | 0 | 0 | 0 | 4 | 4 | 3 |
| 7 | 2 | 45 | M | L | 0,75 | 0,73 | 0,69 | 0,78 | 0 | 0 | 0 |
| 8 | 2 | 46 | M | L | 0,75 | 1,15 | 1,03 | 1,03 | 0 | 0 | 0 |
| 9 | 2 | 72 | M | L | 0,67 | 1,03 | 1,07 | 1,07 | 1 | 0 | 0 |
| 10 | 1 | 46 | M | R | 1 | 0 | 0 | 0 | 4 | 4 | 4 |
| 11 | 1 | 61 | F | R | 1 | 0 | 0 | 0 | 4 | 4 | 4 |
| 12 | 2 | 62 | M | R | 0,75 | 0,95 | 0,92 | 0,89 | 0 | 0 | 0 |
| 13 | 2 | 62 | M | L | 0,83 | 1,06 | 1,07 | 1,04 | 0 | 0 | 0 |
| 14 | 2 | 60 | M | R | 0,58 | 0,43 | 0,42 | 0,99 | 0 | 0 | 0 |
| 15 | 2 | 84 | M | R | 0,75 | 0,53 | 0,61 | 0,63 | 0 | 0 | 0 |
| 16 | 2 | 39 | F | R | 0,25 | 1,05 | 0,87 | 0,91 | 0 | 0 | 0 |
| 17 | 1 | 25 | M | L | 0,5 | 0 | 0 | 0 | 2 | 1 | 1 |
| 18 | 2 | 69 | M | R | 1 | 0,94 | 0,83 | 0,84 | 0 | 0 | 0 |
| 19 | 1 | 34 | M | R | 1 | 0 | 0 | 0 | 3 | 3 | 3 |
| 20 | 2 | 57 | F | R | 0,83 | 0,95 | 1,04 | 0,95 | 0 | 0 | 0 |
| 21 | 1 | 40 | M | R | 1 | 0 | 0,08 | 0,05 | 2 | 1 | 1 |
| 22 | 2 | 45 | F | R | 1 | 0,99 | 0,89 | 0,83 | 0 | 0 | 0 |
| Mean |  | 52 |  |  | 0,75 | 0,58 | 0,59 | 0,59 | 1,2 | 1,1 | 1,1 |
| SD |  | 13 |  |  | 0,37 | 0,48 | 0,50 | 0,47 | 1,7 | 1,6 | 1,6 |

Brain activation maps for each group of patients (for the paretic hand movement *vs*. rest)

| **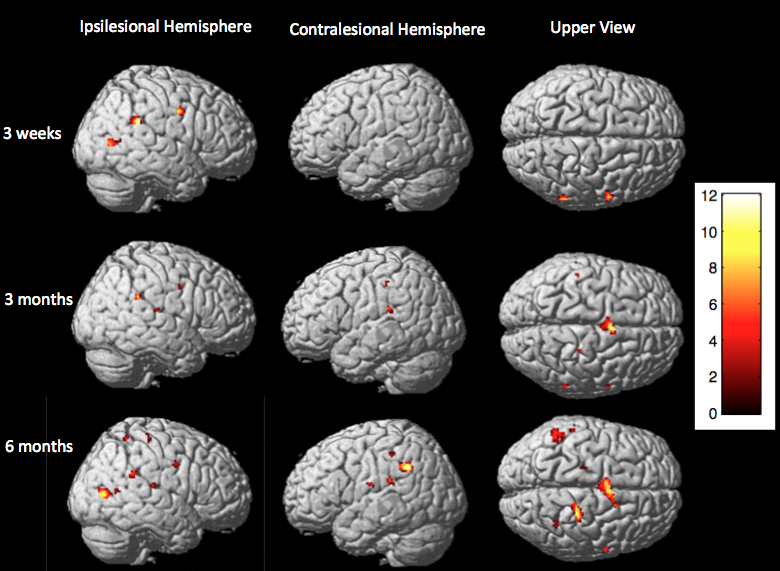** |
| --- |

**Figure S1:** Surface rendering of fMRI activation maps for the contrast “paretic hand movement vs. rest” during the motor task in the severely impaired group. The color bar indicates the t-statistics.

| **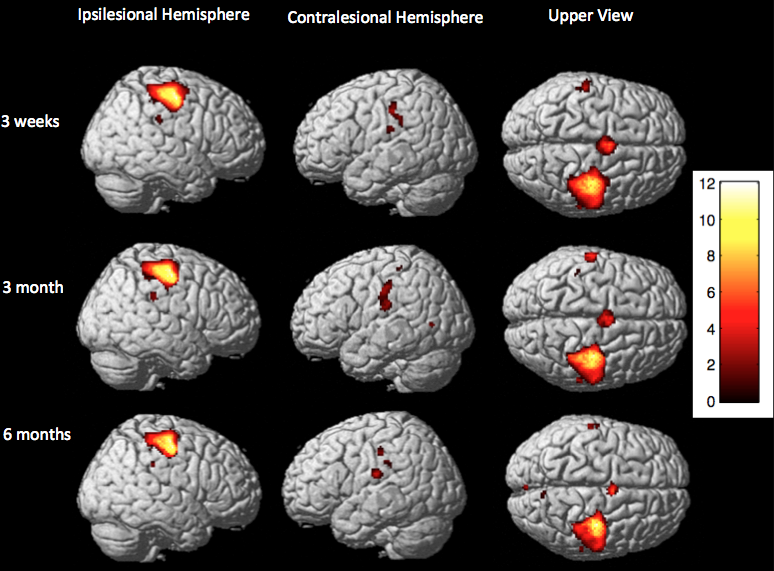** |
| --- |

**Figure S2:** Surface rendering of fMRI activation maps for the contrast “paretic hand movement vs. rest” during the motor task in the mildly impaired group. Color bar indicates the t-statistics.

Cross-sectional analysis on 27 patients at V1

In addition, we re-analyzed the data with five additional patients at V1. Within this cohort (27 patients; mildly impaired n=17 and severely impaired n=10), the results were very similar to those reported in the longitudinal cohort.

In the mildly impaired group compared to the healthy subjects, the abnormal interactions in the IL and CL hemispheres and the IH interactions were similar to those of the longitudinal cohort (22 patients). In the severely impaired group, a similar number of abnormal interactions were found in the IL and CL hemispheres. However, the IH M1-M1 interaction was significantly reduced.

The CST damage in this group of patients (n=27) was highly correlated with the grip strength ratio of the affected hand at V1 (rho: 0.809; 95% CI: 0.663-0.916, p: 0.0001). The global correlations with the grip strength ratio were significant for the functional connectivity indices in M1 in the IL hemisphere for M1-SMA and M1-CER. Compared to the longitudinal cohort, the correlation between IL M1-PMC did not reach significance (rho: 0.321, p: 0.1). Furthermore, the grip strength ratio did not correlate with the functional connectivity values in the CL motor network or with the IH functional connectivity values.
